# Correction to ‘Cooperative Gsx2–DNA binding requires DNA bending and a novel Gsx2 homeodomain interface’

**DOI:** 10.1093/nar/gkae578

**Published:** 2024-06-27

This is a correction to: Jordan A Webb, Edward Farrow, Brittany Cain, Zhenyu Yuan, Alexander E Yarawsky, Emma Schoch, Ellen K Gagliani, Andrew B Herr, Brian Gebelein, Rhett A Kovall, Cooperative Gsx2–DNA binding requires DNA bending and a novel Gsx2 homeodomain interface, *Nucleic Acids Research*, 2024, gkae522, https://doi.org/10.1093/nar/gkae522

The authors failed to cite the program (DNAproDB) used to create the image in Figure 3E.

The caption of Figure 3E has been updated and two references (49,50) were added.

The published article has been updated.

