# Supplementary material for: Cooperative Gsx2–DNA binding requires DNA bending and a novel Gsx2 homeodomain interface
Source: Nucleic Acids Res. 2024 Jun 14;52(13):7987–8002. doi: 10.1093/nar/gkae522 (PMC11260452; doi:10.1093/nar/gkae522)
Supplement: gkae522_Supplemental_File [file gkae522_supplemental_file.pdf]

Supplemental Data

Cooperative Gsx2-DNA Binding Requires DNA Bending and a Novel Gsx2 Homeodomain Interface

Figure S1. AlphaFold predicts full-length Gsx2 to be mostly unstructured except for the HD. Cartoon image of AlphaFold's structural prediction of full-length Gsx2. The structure is colored on a spectrum from red to blue representing the pLDDT value of each residue. The higher the pLDDT, the higher the accuracy of the model. Any residue with a pLDDT > 90 is expected to be modeled with high accuracy, while a pLDDT < 50 is a strong predictor of disorder (35).

Figure S2. The asymmetric unit of Gsx2^{HD}-DNA crystals contain two complexes with a high degree of structural similarity. (A) The asymmetric unit contains two Gsx2^{HD}-DNA complexes. One Gsx2^{HD} molecule is shown in cyan, while the other is in green. DNA for both complexes is grey. (B) An alignment of all 1071 atoms from one Gsx2^{HD}-DNA complex to the other complex gives a resulting RMSD value of 0.265Å. One Gsx2^{HD}-DNA complex is cyan, while the other is green.

Figure S3. Bioinformatic analysis reveals Gsx2 strongly prefers DNA dimer sites with flexible A/T-rich spacer sequences. (A) Tau factor calculations of each sequence used in Figure 5 EMSAs show that all A/T-rich spacer sequences promote increased cooperativity compared to G/C-rich spacers. Significance was determined by one-way ANOVA with Tukey's multiple comparisons test. (B) Measurements of the free probe from EMSAs in Figure 5 reveal no change in Gsx2$^{167\text{-}305}$'s ability to bind the individual DNA sites regardless of the spacer sequence. Bioinformatic DNA shape analyses of these various DNA dimer site sequences showed a narrower minor groove for A/T-rich spacers (C) and increased magnitude of propellor twisting (D) and roll (F) for A/T-rich sequences, all of which are consistent with A/T-rich sequences having

intrinsically more flexibility. No strong discernible pattern was observed with overall helical twist between A/T-rich and G/C-rich spacers (E).

Figure S4. Mutating residues within the modeled Gsx2-Gsx2 binding interface diminish Gsx2's cooperative dimerization on DNA. (A) EMSA comparing WT^{HD} binding on the 7bpS DNA dimer site with three HD mutant constructs; $S212E^{HD}$, $L216E^{HD}$, and $L231E^{HD}$. All mutants decrease cooperativity while maintaining the ability to bind DNA. (B) EMSA comparing WT^{HD} with the same three mutant constructs on the 8bpS DNA dimer site. All constructs bind equally well, with little to no cooperative dimerization observed. Protein concentrations used were 0, 25, 100, and 400 nM.

Figure S5. Triplicate EMSAs comparing WT^{HD} to $I234E^{HD}$ show high reproducibility. EMSA replicates comparing WT^{HD}/$I234E^{HD}$ (A-C) and $WT^{167\text{-}305}$/$I234E^{167\text{-}305}$ (D-F) on both the 7bpS and 8bpS DNA probes. The similarity of I234E binding on the cooperative 7bpS and non-cooperative 8bpS probes demonstrates the significant disruption to Gsx2's ability to dimerize cooperatively on DNA. Protein concentrations of 0, 25, 100, and 400nM were used.

Figure S6. Isothermal titration calorimetry data of Gsx2 203-264 I234E show nearly identical binding characteristics as observed with wildtype Gsx2 203-264. (A) Isotherm of Gsx2 203-264 I234E binding to the 15mer consensus monomer site DNA shows proper stoichiometry with low nanomolar affinity, consistent with wildtype Gsx2 203-264 binding to the same 15mer consensus monomer site DNA.

Figure S1

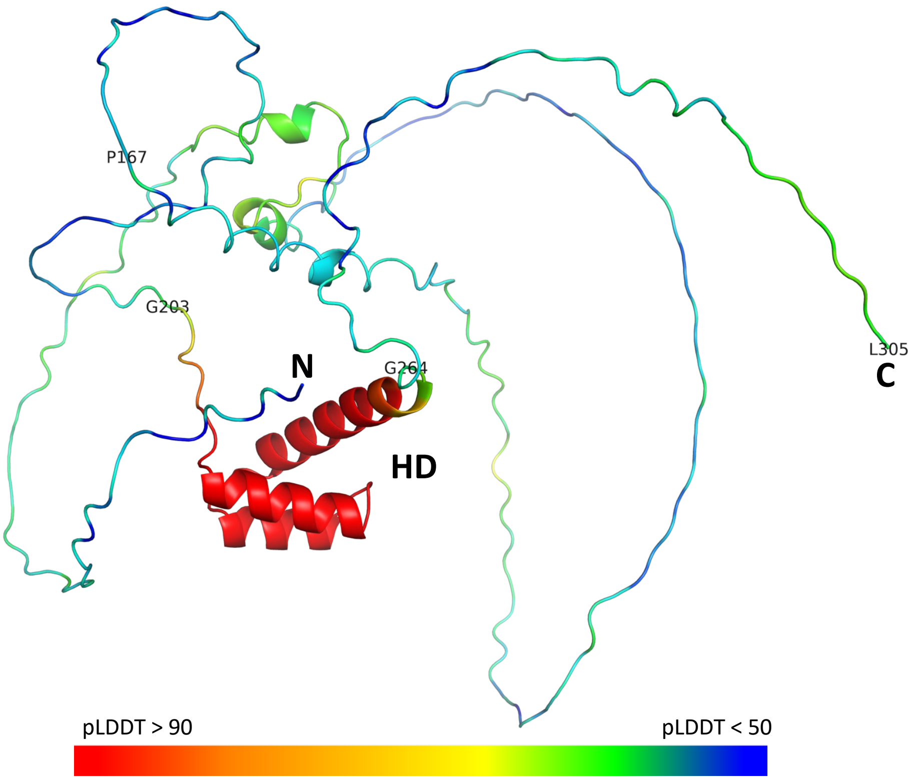

Figure S2

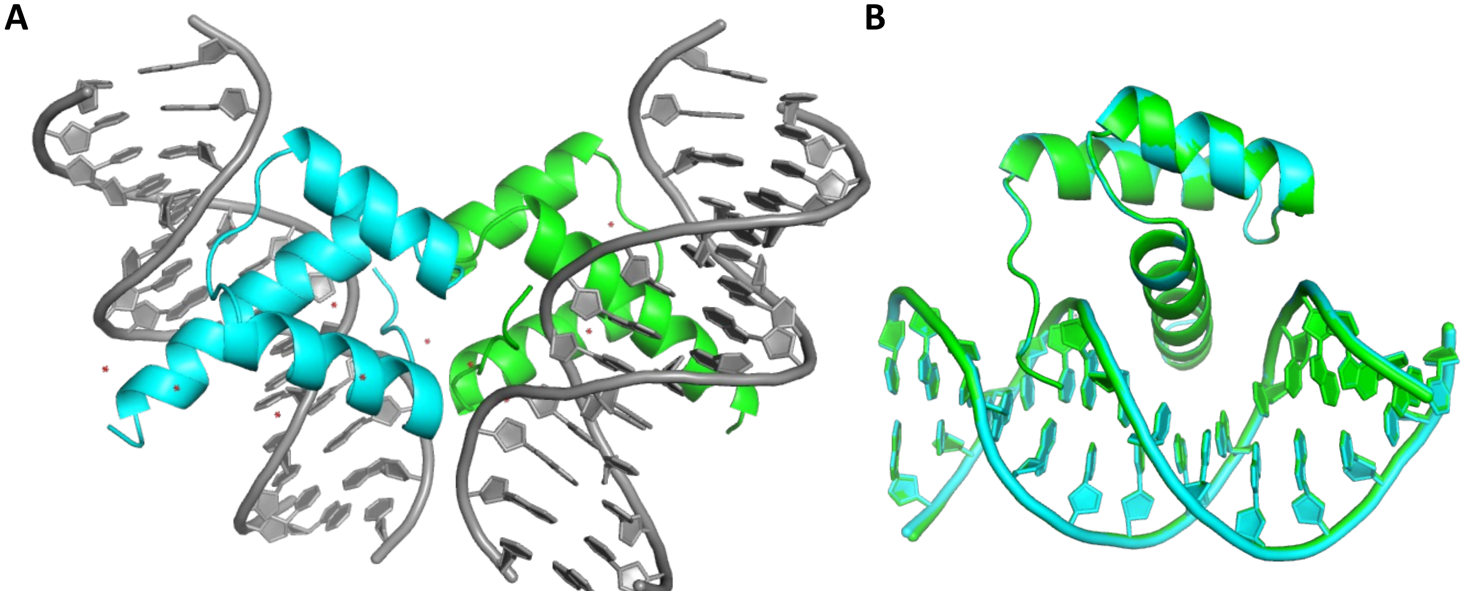

RMSD = 0.265Å (1071 atoms)

Figure S3

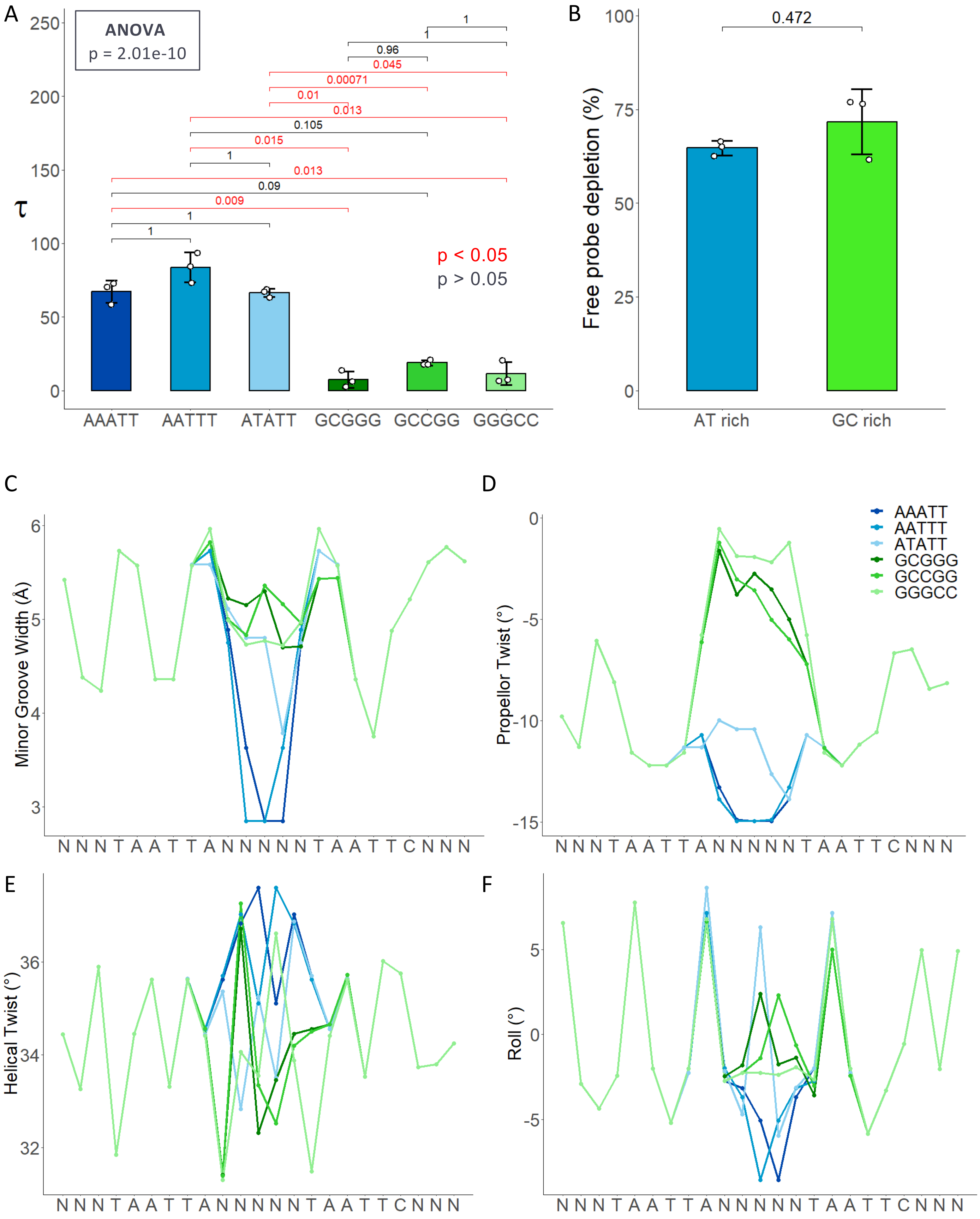
A
ANOVA
p = 2.01e-10
τ
p < 0.05
p > 0.05
AAATT
AATTT
ATATT
GCGGG
GCCGG
GGGCC
B
Free probe depletion (%)
0.472
AT rich
GC rich
C
Minor Groove Width (Å)
D
Propellor Twist (°)
AAATT
AATTT
ATATT
GCGGG
GCCGG
GGGCC
E
Helical Twist (°)
F
Roll (°)
N N N T A A T T A N N N N N N T A A T T C N N N

Figure S4

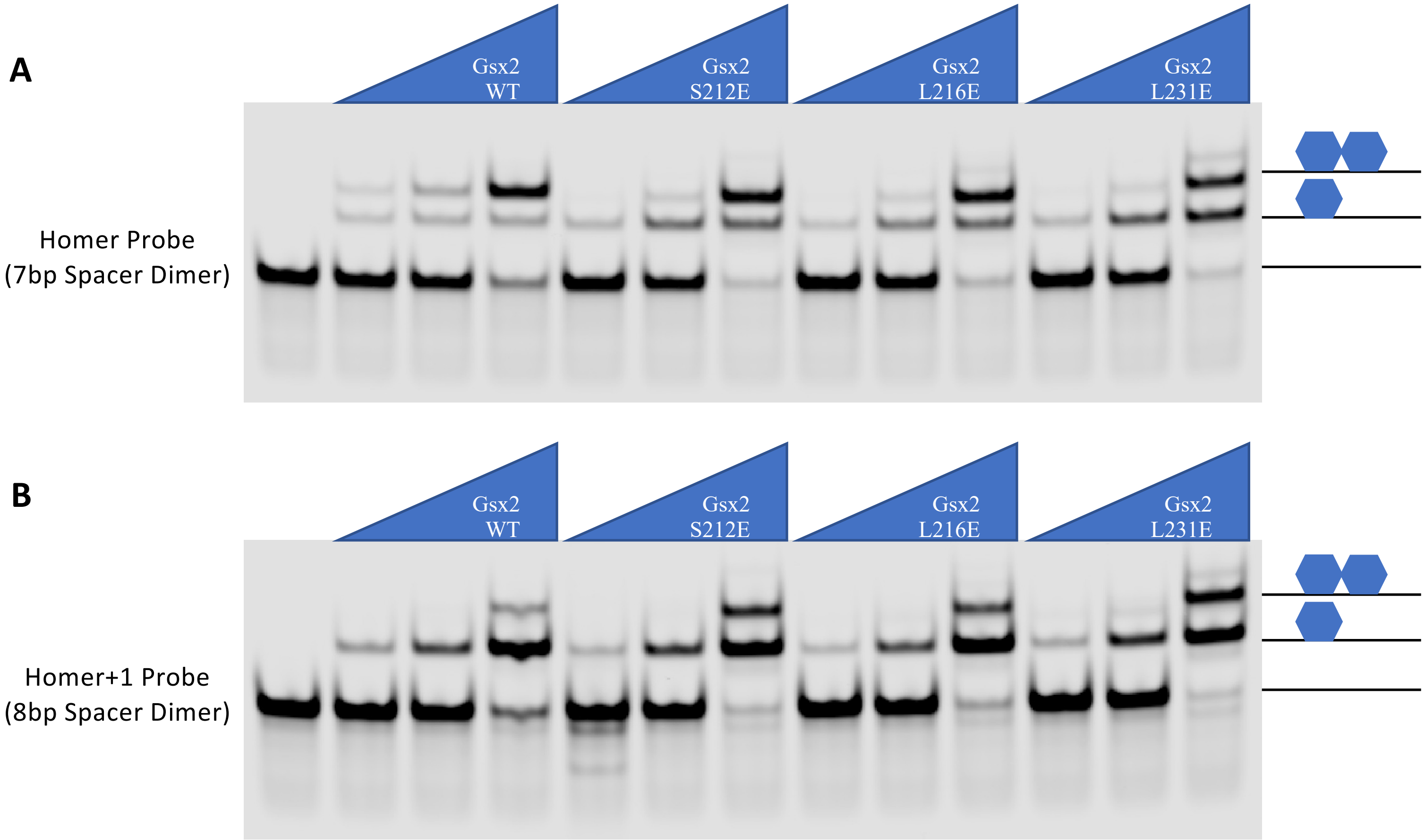
A
Gsx2
WT
Gsx2
S212E
Gsx2
L216E
Gsx2
L231E
Homer Probe
(7bp Spacer Dimer)
B
Gsx2
WT
Gsx2
S212E
Gsx2
L216E
Gsx2
L231E
Homer+1 Probe
(8bp Spacer Dimer)

Figure S5

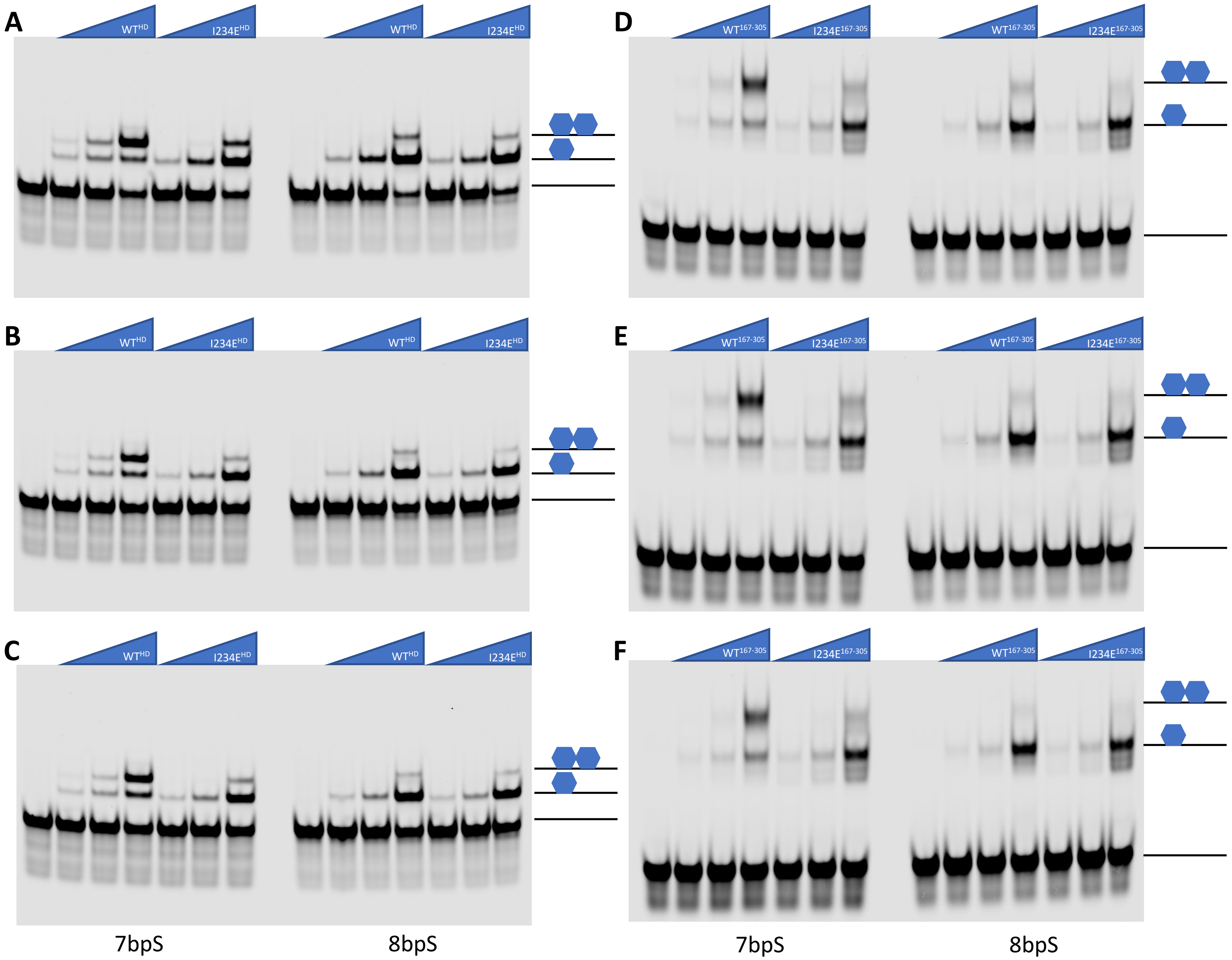

Figure S6

TAATTA Motif

A

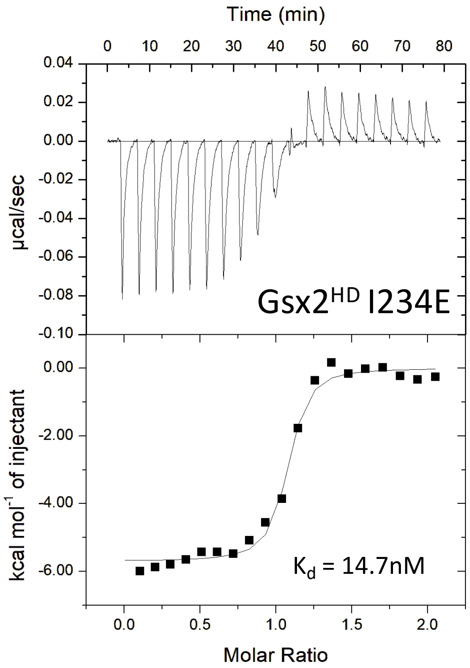

Table S1. Curves+ analysis of our solved Gsx2-DNA structure.

```
*************************************        **************
****   CURVES+ Version 3.0nc 09/2016   ***        *   28 Jun 22   *
*************************************        **************
```

FILE : 8EML.pdb ftop :
LIS : output LIB : standard
ibld : sol :
BACK : P

WBACK : 2.90 WBASE : 3.50 RVFAC : 7.50

isym : 1 itst : 0 itnd : 0 itdel : 1 itbkt : 0
NAXLIM: 1

CIRC : F LINE : F ZAXE : F FIT : T test : F
ions : F REFO : F axfrm : F frames: F

LS fitting of standard bases ...RMS max = 0.039

Strands = 2 Atoms = 574 Units = 28

Combined strands have 14 levels ...

Strand 1 has 14 bases (5'-3'): GAGCTAATTAAAGC
Strand 2 has 14 bases (3'-5'): CTCGATTAATTTCG

(A) BP-Axis

	Xdisp	Ydisp	Inclin	Tip	Ax-bend
1) G 2-C 15	0.14	-1.73	8.4	9.5	---
2) A 3-T 14	-0.52	0.15	9.5	1.5	2.0
3) G 4-C 13	-0.11	-0.07	9.9	3.7	2.3
4) C 5-G 12	-0.09	-0.81	4.6	-0.7	2.2
5) T 6-A 11	-0.99	-0.87	11.4	-1.1	1.9
6) A 7-T 10	-0.93	0.26	7.4	-1.0	2.2
7) A 8-T 9	-0.76	0.16	2.5	-3.2	1.7
8) T 9-A 8	0.13	-0.84	-3.8	-3.4	1.7
9) T 10-A 7	-0.90	-1.01	-0.2	-2.7	1.9
10) A 11-T 6	0.42	-0.00	6.2	1.9	2.0
11) A 12-T 5	-0.70	-0.01	0.8	1.9	1.8
12) A 13-T 4	-0.71	-0.23	0.3	-0.7	1.5
13) G 14-C 3	-0.73	-0.25	4.7	3.9	1.8
14) C 15-G 2	-1.00	-1.30	8.2	1.8	2.1

Average: -0.48 -0.47 5.0 0.8 Total bend = 19.9 (2 to 13)

(B) Intra-BP parameters

Strands 1-2	Shear	Stretch	Stagger	Buckle	Propel	Opening
1) G 2-C 15	-0.66	-0.51	-0.80	-1.9	-14.4	10.2
2) A 3-T 14	0.30	0.05	-0.03	13.9	-16.4	1.3
3) G 4-C 13	0.99	-0.24	0.04	8.0	-8.3	-1.3

4) C 5-G 12	-0.38	-0.14	0.61	-6.2	-11.7	3.3
5) T 6-A 11	-0.43	0.21	-0.18	1.9	-16.1	3.6
6) A 7-T 10	0.40	-0.44	-0.27	-2.6	-8.9	5.3
7) A 8-T 9	0.29	-0.15	-0.08	6.2	-15.9	1.3
8) T 9-A 8	-0.16	0.00	0.23	2.7	-10.8	9.8
9) T 10-A 7	-0.06	0.04	-0.29	7.3	-9.3	6.7
10) A 11-T 6	0.17	-0.33	-0.24	2.1	-4.0	8.6
11) A 12-T 5	-0.08	-0.16	0.30	2.4	-13.3	-1.9
12) A 13-T 4	-0.23	-0.33	0.19	-1.9	-5.9	1.0
13) G 14-C 3	0.16	0.07	0.14	8.8	-5.9	-2.3
14) C 15-G 2	-0.24	-0.06	-0.28	12.1	-7.0	-1.9
Average:	0.01	-0.14	-0.05	3.8	-10.6	3.1

(C) Inter-BP	Shift	Slide	Rise	Tilt	Roll	Twist	H-Ris	H-Twi
1) G 2/A 3	0.04	1.91	2.82	-4.7	-0.8	40.3	3.05	40.6
2) A 3/G 4	0.21	-0.29	3.46	-2.3	10.0	33.1	3.34	34.2
3) G 4/C 5	0.02	-0.68	3.56	-5.9	1.6	29.4	3.44	29.7
4) C 5/T 6	-0.41	-0.31	2.98	8.1	6.0	33.8	2.90	34.4
5) T 6/A 7	0.01	0.56	3.54	-1.0	7.9	40.8	3.57	41.5
6) A 7/A 8	-0.24	-0.22	3.08	-1.9	0.8	31.9	3.02	31.9
7) A 8/T 9	1.12	-0.89	3.36	-2.9	-0.9	27.6	3.44	27.6
8) T 9/T 10	-0.18	-0.34	3.08	6.8	-1.8	35.5	3.11	35.7
9) T 10/A 11	1.59	0.99	3.46	6.7	4.5	38.5	3.51	38.6
10) A 11/A 12	-1.31	0.21	3.34	-7.0	0.4	34.1	3.38	34.2
11) A 12/A 13	-0.03	-0.42	3.32	-2.2	-2.9	38.0	3.32	38.0
12) A 13/G 14	0.23	-0.07	3.24	1.7	6.6	33.9	3.21	34.1
13) G 14/C 15	0.37	-1.02	3.19	1.0	3.0	29.0	2.99	29.3
Average:	0.11	-0.04	3.26	-0.3	2.6	34.3	3.25	34.6

(D) Backbone Parameters

Strand 1	Alpha	Beta	Gamma	Delta	Epsil	Zeta	Chi	Phase	Ampli	Puckr
1) G 2	----	90.4	46.8	139.1	-149.5	-166.0	-64.3	158.0	38.2	C2'en
2) A 3	-44.8	117.1	67.5	117.9	-171.9	-116.9	-110.4	126.2	33.6	C1'ex
3) G 4	4.1	144.6	-0.5	143.6	-140.3	-145.7	-99.7	152.9	44.5	C2'en
4) C 5	-63.3	131.5	58.7	79.2	-174.9	-74.2	-160.6	53.9	44.1	C4'ex
5) T 6	-71.3	-174.1	66.8	140.8	-166.4	-120.8	-113.6	161.3	34.5	C2'en
6) A 7	39.0	172.4	-52.3	156.2	179.8	-100.7	-99.7	-157.8	35.5	C3'ex
7) A 8	-42.2	175.4	29.9	141.5	-155.4	-122.9	-96.6	160.5	39.8	C2'en
8) T 9	62.6	-129.3	-112.2	124.0	-160.2	-80.5	-147.1	145.5	16.1	C2'en
9) T 10	-70.2	176.6	56.6	126.9	-166.2	-93.5	-108.7	137.6	35.8	C1'ex
10) A 11	-58.6	167.9	46.3	140.2	-97.2	166.3	-95.9	144.2	48.4	C2'en
11) A 12	-74.3	136.3	46.1	149.1	-129.8	-136.1	-111.1	157.1	46.7	C2'en
12) A 13	-64.1	149.2	34.6	102.9	-177.7	-93.1	-131.1	95.8	35.0	O1'en
13) G 14	-52.4	171.3	50.7	137.3	-168.9	-106.6	-106.5	153.3	37.1	C2'en
14) C 15	-69.4	156.0	80.2	113.2	----	----	-138.0	116.8	47.7	C1'ex

Strand 2	Alpha	Beta	Gamma	Delta	Epsil	Zeta	Chi	Phase	Ampli	Puckr
1) C 15	-17.9	155.9	37.1	146.6	----	----	-88.6	-173.1	33.0	C3'ex
2) T 14	7.5	138.3	3.6	124.7	-177.8	-133.7	-111.0	133.8	36.7	C1'ex
3) C 13	-40.6	158.6	47.7	127.9	-163.7	-133.9	-126.2	136.4	40.0	C1'ex

4)	G	12	-49.0	164.9	40.1	123.5	-173.3	-118.9	-117.4	129.8	37.2	C1'ex
5)	A	11	-0.1	143.4	13.4	150.5	-173.6	-117.2	-89.1	169.6	41.8	C2'en
6)	T	10	19.4	155.9	-14.9	150.9	-165.0	-149.7	-92.8	170.5	42.4	C2'en
7)	T	9	-64.2	176.9	57.2	118.6	-175.5	-116.9	-126.6	126.0	36.4	C1'ex
8)	A	8	-67.1	179.0	55.1	111.7	178.9	-91.3	-127.6	111.9	32.5	C1'ex
9)	A	7	-55.9	147.9	38.1	141.3	170.1	-91.3	-105.7	175.0	32.1	C2'en
10)	T	6	-59.4	-178.9	43.3	141.6	-121.9	-179.3	-99.5	148.3	45.7	C2'en
11)	T	5	-60.8	167.2	56.7	120.9	-179.6	-85.0	-121.4	128.2	38.2	C1'ex
12)	T	4	-64.3	172.8	62.8	131.7	-171.2	-104.3	-115.2	152.5	32.9	C2'en
13)	C	3	-55.6	-177.3	45.5	136.5	-164.8	-109.8	-128.3	152.1	35.0	C2'en
14)	G	2	----	125.6	-2.4	95.4	-175.0	-75.5	-159.1	53.9	30.7	C4'ex

(E) Groove parameters

Level			W12	D12	W21	D21
1.5						
2.0	A	3				
2.5						
3.0	G	4	8.1	4.1		
3.5			7.5	4.8		
4.0	C	5	6.8	5.0		
4.5			7.1	4.8	10.8	6.5
5.0	T	6	7.2	4.4	10.4	6.9
5.5			6.9	4.7	10.6	6.9
6.0	A	7	6.4	5.0	11.0	6.5
6.5			6.2	4.8	10.6	6.2
7.0	A	8	5.9	4.5	10.4	5.8
7.5			5.5	5.1	11.5	5.7
8.0	T	9	5.2	5.3	12.8	5.0
8.5			5.5	5.0	13.3	5.3
9.0	T	10	5.8	4.6	13.4	5.7
9.5			4.6	5.9	13.5	5.1
10.0	A	11	3.8	6.9	12.9	3.9
10.5			4.7	6.1	12.2	4.2
11.0	A	12	6.0	5.0		
11.5			6.8	4.9		
12.0	A	13				
12.5						
13.0	G	14				
13.5						

Table S2. Oligonucleotides used for EMSAs, ITC, X-ray crystallography, and site-directed mutagenesis.

**EMSA**	
AAATT	5'-TCCAAC**TAAT**TAAAATT**TAAT**TCG*TAGTGCGGGCGTGGCT*-3'
AATTT	5'-TCCAAC**TAAT**TAAATTT**TAAT**TCG*TAGTGCGGGCGTGGCT*-3'
ATATT	5'-TCCAAC**TAAT**TAATATT**TAAT**TCG*TAGTGCGGGCGTGGCT*-3'
GCGGG	5'-TCCAAC**TAAT**TAGCGGG**TAAT**TCG*TAGTGCGGGCGTGGCT*-3'
GCCGG	5'-TCCAAC**TAAT**TAGCCGG**TAAT**TCG*TAGTGCGGGCGTGGCT*-3'
GGGCC	5'-TCCAAC**TAAT**TAGGGCC**TAAT**TCG*TAGTGCGGGCGTGGCT*-3'
Gsx2 7bpS	5'-TCCAAC**TAAT**TAAAATT**TAAT**TCG*TAGTGCGGGCGTGGCT*-3'
Gsx2 8bpS	5'-TCCAAC**TAAT**TAAAGATT**TAAT**TCG*TAGTGCGGGCGTGGCT*-3'
Linker for Probe	5'-IRDye700-*AGCCACGCCCGCACTA*-3'
**Isothermal Titration Calorimetry**	
Consensus Site Forward	5'-TGAGC**TAATTA**AAGC-3'
Consensus Site Reverse	5'-CTCG**ATTAAT**TTCGA-3'
Common Q50 Site Forward	5'-TGAGC**TAATGG**AAGC-3'
Common Q50 Site Reverse	5'-CTCG**ATTACC**TTCGA-3'
**X-ray Crystallography**	
Consensus Site Forward	5'-TGAGC**TAATTA**AAGC-3'
Consensus Site Reverse	5'-CTCG**ATTAAT**TTCGA-3'
**Site-Directed Mutagenesis**	
Gsx2 S212E Forward	5'-GAGGACAGCGTTTACCGAGACGCAGCTCCTGGAGC-3'
Gsx2 S212E Reverse	5'-GCTCCAGGAGCTGCGTCTCGGTAAACGCTGTCCTC-3'
Gsx2 L216E Forward	5'-CAGCACGCAGCTCGAGGAGCTGGAGCGA-3'
Gsx2 L216E Reverse	5'-TCGCTCCAGCTCCTCGAGCTGCGTGCTG-3'
Gsx2 L231E Forward	5'-CCAATATGTACCTGTCCCGAGAGCGGAGAATCGAGATCGC-3'
Gsx2 L231E Reverse	5'-GCGATCTCGATTCTCCGCTCTCGGGACAGGTACATATTGG-3'
Gsx2 I234E Forward	5'-GTCCCGACTCCGGAGAGAGGAGATCGCGACATACC-3'
Gsx2 I234E Reverse	5'-GGTATGTCGCGATCTCCTCTCTCCGGAGTCGGGAC-3'

*Binding sites are bold and underlined. Linker sequences are italicized.
